# Tropical cyclone dataset for a high-resolution global nonhydrostatic atmospheric simulation

**DOI:** 10.1016/j.dib.2023.109135

**Published:** 2023-04-11

**Authors:** Daisuke Matsuoka, Chihiro Kodama, Yohei Yamada, Masuo Nakano

**Affiliations:** a Center for Earth Information Science and Technology (CEIST), Research Institute for Value-Added-Information Generation (VAiG), Japan Agency for Marine-Earth Science and Technology (JAMSTEC) 3173-25 Showa-machi, Kanazawa-ku, Yokohama, Kanagawa 236-0001 Japan; b Research Center for Environmental Modeling and Application (CEMA), Research Institute for Global Change (RIGC), Japan Agency for Marine-Earth Science and Technology (JAMSTEC) 3173-25 Showa-machi, Kanazawa-ku, Yokohama, Kanagawa 236-0001 Japan; c Typhoon Science and Technology Research Center (TRC), Institute for Multidisciplinary Sciences (IMS), Yokohama National University 79-1 Tokiwadai, Hodogaya-ku, Yokohama, Kanagawa 240-0067 Japan

**Keywords:** Cyclogenesis, Precursor, Image recognition, Machine learning, AI

## Abstract

This dataset is a time series of tropical cyclones simulated using the high-resolution Nonhydrostatic Icosahedral Atmospheric Model (NICAM). By tracking tropical cyclones from 30 years of simulation data, 2,463 tracks that include the life stages of precursors (pre-TCs), tropical cyclones (TCs), and post-tropical cyclones (post-TCs), if any, were extracted. Each track data includes the time, latitude, longitude, maximum wind speed, minimum pressure, elapsed time since onset, and life-stage label of the tropical cyclone. The numbers of steps (6 h) for pre-TCs, TCs, and post-TCs were 45,288, 55,206, and 37,312, respectively. The dataset for each step also consists of atmospheric field data of multiple physical quantities, such as outgoing longwave radiation at the top-of-the-atmosphere, sea level pressure, sea surface temperature, specific humidity at 600 hPa, and zonal and meridional winds at 850 and 200 hPa over a 1000 km^2^ area that includes a tropical cyclone at its center. This dataset can be used to develop machine-learning models for the detection, intensity prediction, and cyclogenesis prediction of tropical cyclones.

**Specifications Table**

**Table:** 

Subject	Computers in Earth Sciences
Specific subject area	Pattern recognition of tropical cyclone
Type of data	Tables (track data) Unformatted binary data (simulation data)
How the data were acquired	A high-resolution global nonhydrostatic atmospheric simulation model (NICAM) and a tropical cyclone tracking algorithm. The K computer was used for numerical simulation.
Data format	Raw Analyzed
Description of data collection	Tropical cyclones (TCs) were tracked and cropped from 6-h climate simulation data using a TC tracking algorithm. The simulation was performed for 30 years with external forcings from 1979 to 2008. The atmospheric field data were a rectangular area of approximately 1000 km^2^ (64 ×64 grid) that included the center of the tropical cyclone.
Data source location	• Institution: JAMSTEC • City/Town/Region: Yokohama • Country: Japan • Latitude and longitude (and GPS coordinates, if possible) for collected samples/data: not applicable
Data accessibility	Repository name: Mendeley Data Data identification number: 10.17632/xtvvkfvycr.1 Direct URL to data: https://data.mendeley.com/datasets/xtvvkfvycr/1

## Value of the Data

• This dataset provides a time series of simulated tropical cyclones with a long time period of 30 years, a high spatial resolution of 14 km, and 8 representative physical quantities.
• Atmospheric scientists can use this dataset to develop machine-learning models for detection, intensity prediction, and cyclogenesis prediction of tropical cyclones.
• Computer scientists can use this dataset to develop machine-learning models for image pattern prediction and spatio-temporal prediction as benchmark data.

## 1 Objective

The NICAM tropical cyclone (TC) dataset aims to develop data-driven models for tropical cyclone detection, intensity estimation, and development prediction. Tropical cyclones bring heavy rains and strong winds that can cause serious damage to human life. Therefore, accurately predicting tropical cyclones is one of the most important topics in meteorology. In conventional weather forecasting, process-driven approach using numerical simulation models has been mainly used. However, it is difficult to accurately forecast extreme phenomena such as tropical cyclones, and there are growing expectations for a data-driven approach using machine learning. The use of this data as benchmark data on tropical cyclone forecasting will promote the deployment of machine learning in the field of meteorology. On the other hand, note that the accuracy of the learning model constructed using this data would be reduced when applied to other simulation or observation data. In addition, it cannot be applied directly to the forecasting or estimation of other phenomena (e.g., rainfall and temperature).

## 2 Data Description

**Fig. 1:**
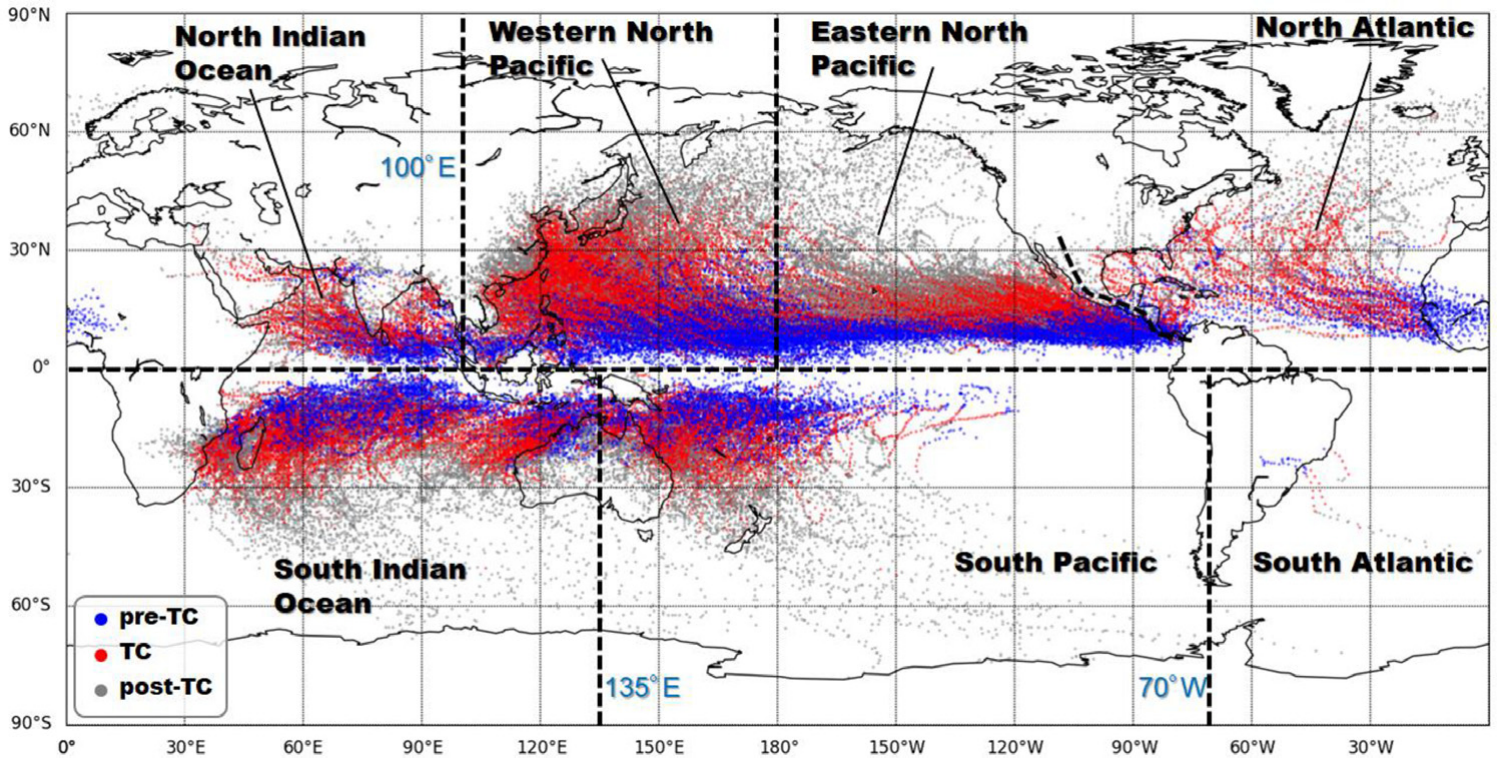
Tracking results of tropical cyclone precursors (pre-TCs; blue dots), TCs (red dots), and post-TCs (gray dots) during 1979–2008 from NICAM.

The NICAM tropical cyclone (TC) dataset was produced by implementing the TC tracking algorithm [1,2] with 30 years of climate simulation data from the high-resolution global Nonhydrostatic Icosahedral Atmospheric Model (NICAM; Kodama et al. 2015). The simulation was performed using external forcings from to 1979–2008. The datasets are available from Mendeley Data at https://data.mendeley.com/datasets/xtvvkfvycr/1. Fig. 1 illustrates the tracks of 2463 TCs that occurred during the 30-year study period. Tracks include TCs as well as their precursors (pre-TCs, before becoming TCs) and post-TCs.

**Fig. 2:**
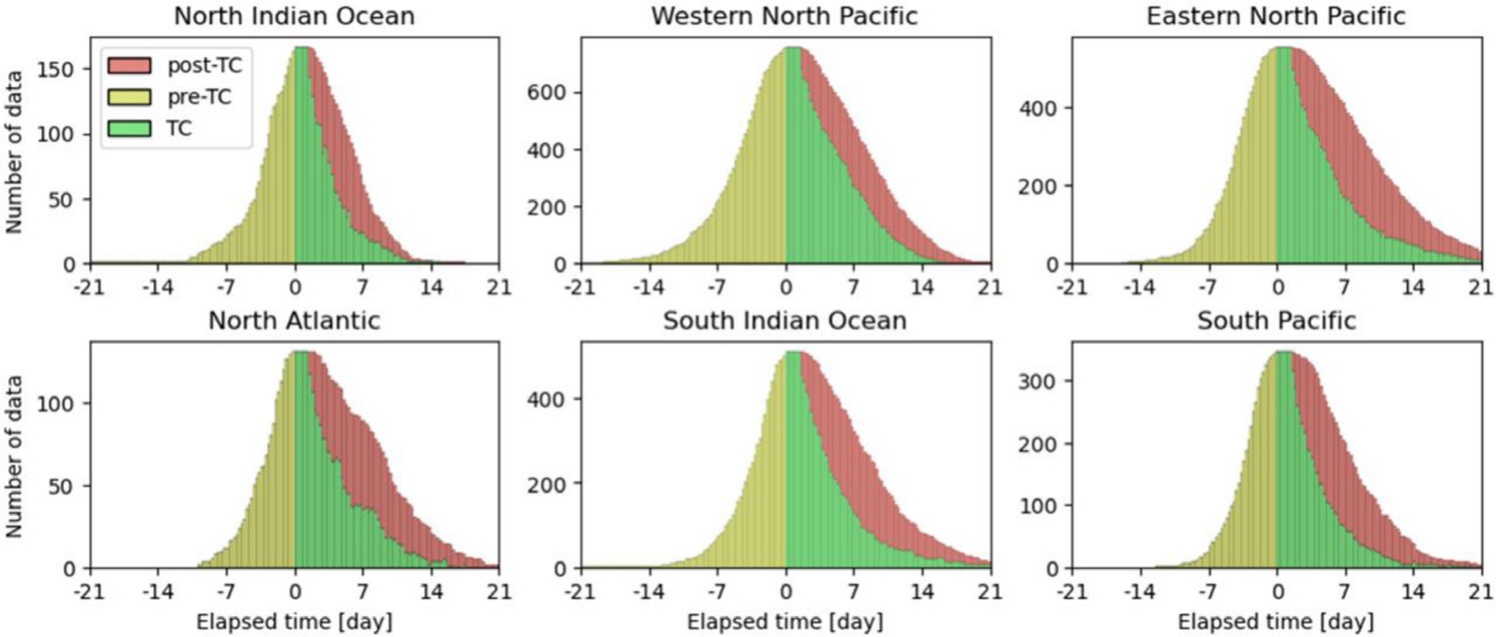
Number of data (pre-TCs, TCs, and post-TCs) of each elapsed time frame (bin width of 6 h) based on tropical cyclone (TC) genesis in each basin. The basin is based on the location at the time of genesis.

**Fig. 3:**
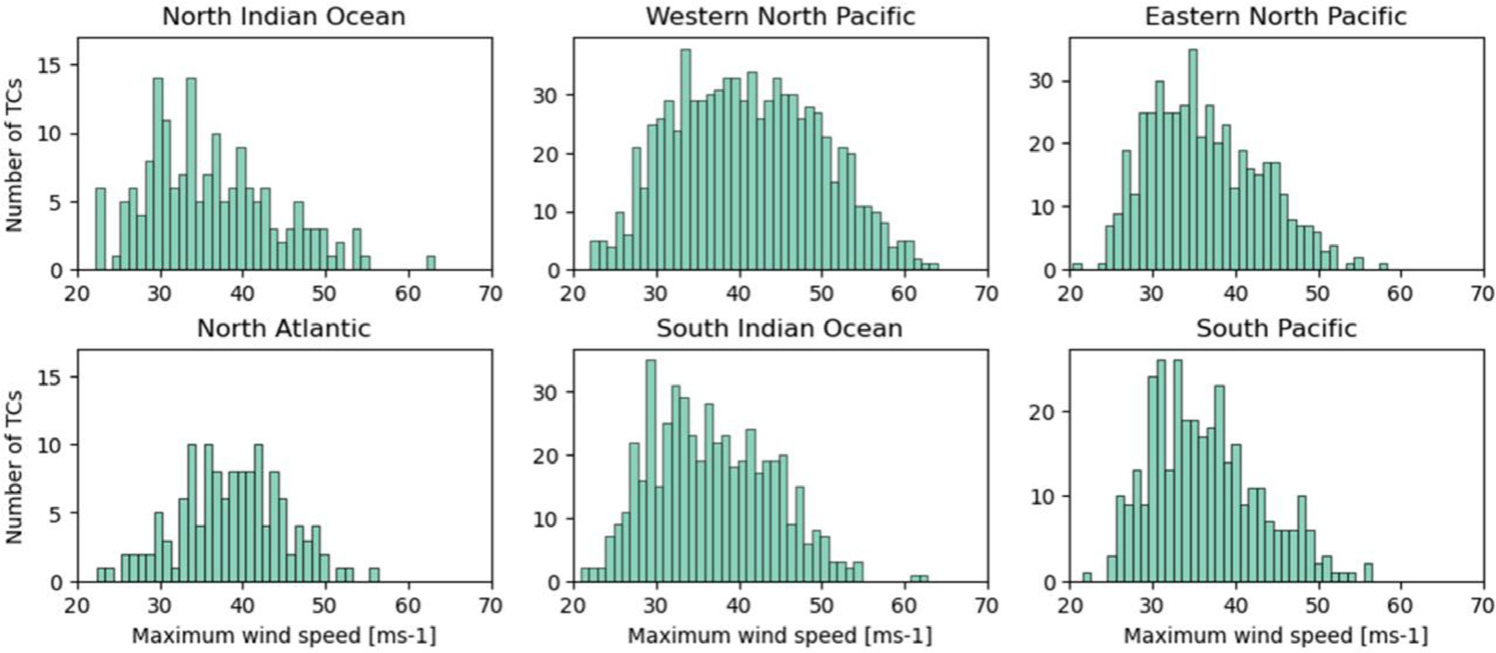
Histogram of lifetime-maximum wind speed at 10 m in each basin (bin width of 1 ms^−1^).

**Fig. 4:**
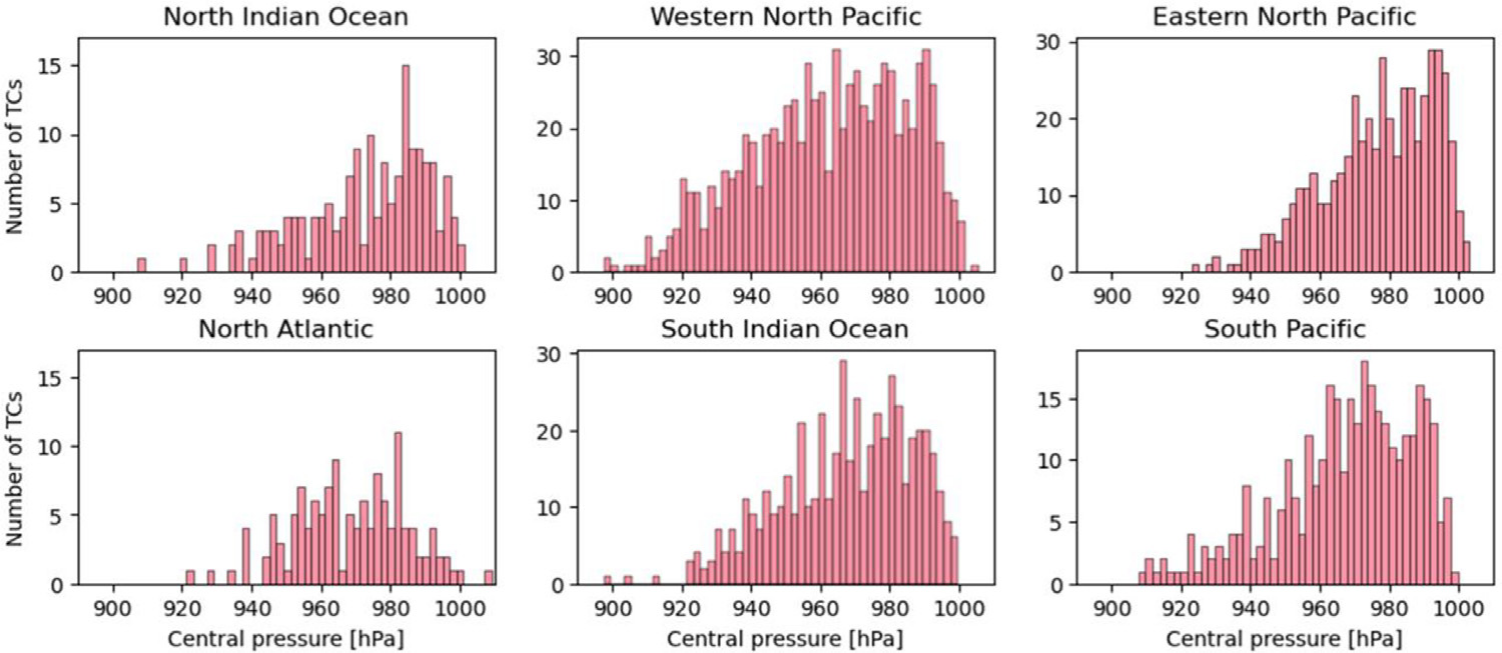
Histogram of lifetime-minimum central pressure in each basin (bin width of 2 hPa).

The numbers of snapshots of pre-TCs, TCs, and post-TCs clipped every 6 h were 45,288, 55,206, and 37,312, respectively. Fig. 2 shows the number of data points for each ocean basin, excluding the South Atlantic, where there are few cases. In addition, histograms of the lifetime-maximum wind speed at the 10 m level and the lifetime-minimum central pressure for each TC track for each basin are shown in Figs. 3 and 4. The number of TC genesis events, tracks, and intensities for each ocean basin in the simulation are in good agreement with observations [3].

This dataset consisted of TC track data (CSV format) and atmospheric field data (NPZ format), as shown in the directory structure in Table 1. All track data are stored in one folder. Atmospheric field data are divided into folders by year. Within the folders for each year, there are subfolders for each physical quantity. The details regarding each data point are described in the following sections.

**Table 1:** Overview of the dataset directory.

### 2.1 Track Data

The track data comprised the date and time (in UTC), longitude (deg), latitude (deg), maximum wind speed at the 10 m level (ms^−1^), central pressure (hPa), elapsed time based on genesis (h), and life-stage label (pre-TC, TC, or post-TC). The transition from pre-TC to TC (genesis of TC) and from TC to post-TC (dissipation of TC) is defined by criteria such as wind speed, relative vorticity, temperature, and duration, as outlined in the ‘TC Tracking’ section. The elapsed time was calculated based on the occurrence of a TC, with the origin point of cyclogenesis and negative values indicating the pre-TC period.

Each TC track data is stored in a separate file (CSV format) with a header. The file name format is “[YYYY][MM][DD][HH]_lon[longitude]_lat[latitude].csv” using the information of date, time, latitude, and longitude at the time of TC occurrence. Here, [latitude] must be a positive value and is distinguished by adding “n” for the Northern Hemisphere and “s” for the Southern Hemisphere (e.g., "lat30s" for 30° south latitude). For ease of handling, longitude is indicated in the range from 0°E to 360°E. An example of TC track data is shown in Table 2.

**Table 2:** Example of tropical cyclone (TC) track data.

Time [HHzDDMMMYYYY]	Longitude [deg]	Latitude [deg]	Maximum 10 m wind speed [ms^−1^]	Central pressure [hPa]	Elapsed time based on genesis [h]	Life-stage label (pre-TC: 0, TC: 1, post-TC: 2)
12Z09JUL1982	218.281	10.037	13.9	1007	−24	0
18Z09JUL1982	216.454	9.615	16.8	1006.7	−18	0
00Z10JUL1982	215.469	10.459	16.2	1005.5	−12	0
06Z10JUL1982	213.501	10.74	16.2	1005.3	−6	0
12Z10JUL1982	211.954	10.88	20.8	1000.8	0	1
18Z10JUL1982	210.408	11.021	19.0	1003.3	6	1
00Z11JUL1982	209.142	11.302	21.2	1002.1	12	1
06Z11JUL1982	207.736	11.583	24.5	998.75	18	1
12Z11JUL1982	206.752	11.724	23.4	999.34	24	1
18Z11JUL1982	205.627	11.865	25.8	998.04	30	1
00Z12JUL1982	204.924	11.724	23.8	996.21	36	1
06Z12JUL1982	204.503	12.286	26.9	994.43	42	1
12Z12JUL1982	203.659	12.849	24.2	997.91	48	1
18Z12JUL1982	202.956	13.411	30.0	992.03	54	1
00Z13JUL1982	202.394	14.255	32.7	990.79	60	2
06Z13JUL1982	201.55	15.239	29.4	994.14	66	2
12Z13JUL1982	200.285	16.083	22.8	1004.0	72	2

### 2.2 Atmospheric Field Data

**Fig. 5:**
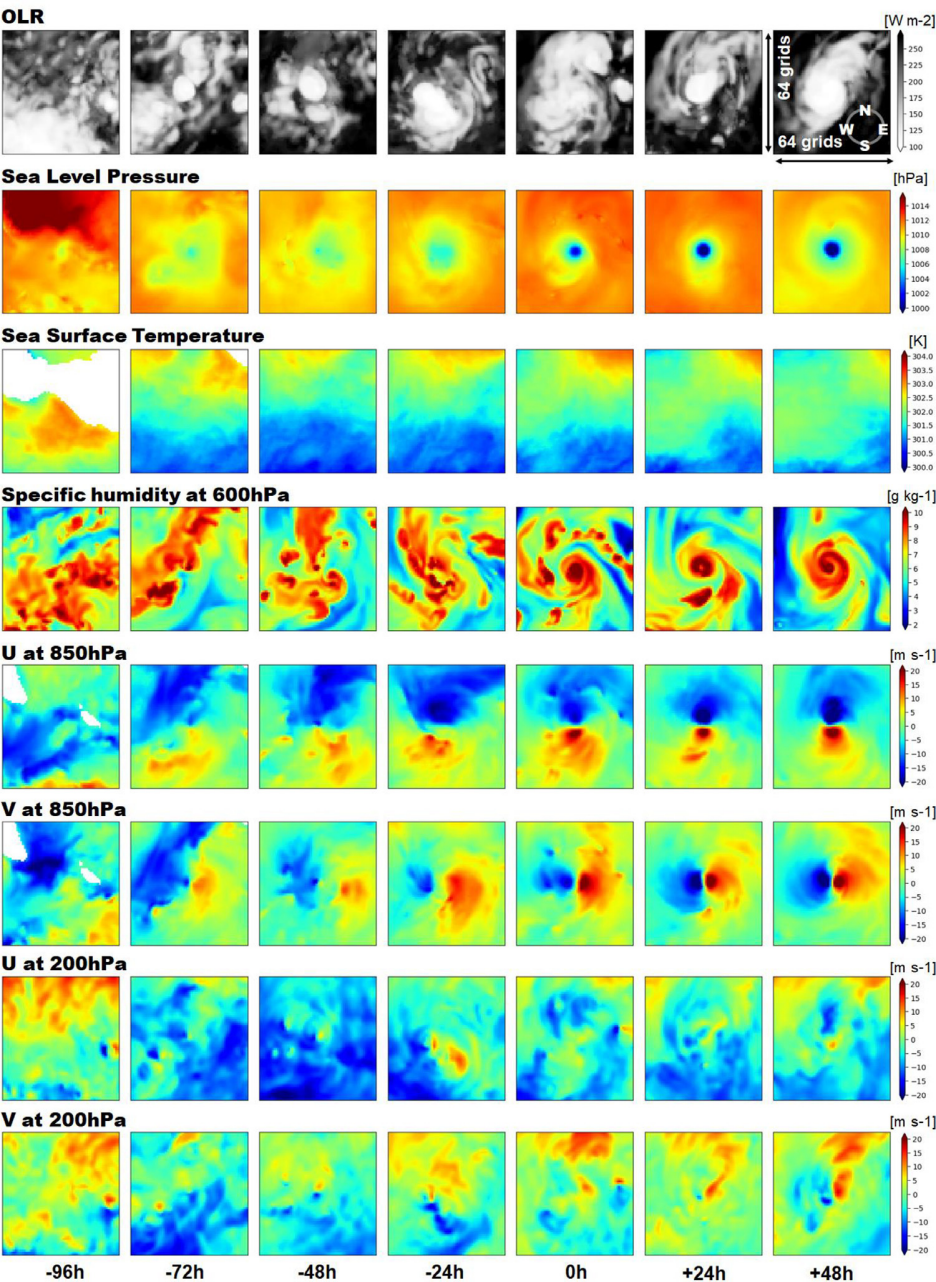
Examples of atmospheric field data for a tropical cyclone. Each image is composed of a 64 × 64 grid, corresponding to an area of approximately 1000 km × 1000 km in actual scale.

The atmospheric field data consist of various physical quantities in a small rectangular region that covers each TC detected as pre-TC, TC, or post-TC in the track data. The grid size of the cropped area is 64 ×64, which is approximately 1000 km × 1000 km in actual scale (horizontal resolution at the equator of the NICAM is approximately 14 km). The physical quantities available online are outgoing longwave radiation (OLR) at the top-of-atmosphere, sea level pressure (SLP), sea surface temperature (SST), specific humidity at 600 hPa, and zonal and meridional winds at 850 and 200 hPa (Table 3). These are physical quantities and altitude levels that are related to the occurrence and development of TCs. Fig. 5 shows the physical quantities of an example TC from 96 h before to 48 h after its onset.

**Table 3:** Overview of each physical quantity.

	Pressure level	Unit	Abbreviation
Outgoing longwave radiation	–	W/m2	OLR
Sea level pressure	–	Pa	SLP
Sea surface temperature	–	K	SST
Specific humidity	600 hPa	g/kg	QV600
Zonal wind	850/200 hPa	m/s	U850/U200
Meridional wind	850/200 hPa	m/s	V850/V200

The file format of the atmospheric field data is NPZ, which is array data for numpy using gzip compression; metadata are not supported. Numpy is an extension module of Python, the most popular programming language used in machine learning, which efficiently performs numerical computations. Missing values such as SST on land are filled with “np.nan”. Therefore, these data can be used immediately to build models for TC detection, intensity estimation, and development prediction using machine learning [4,5]. The file name format is “[var]_[YYYY][MM][DD][HH]_lon[longitude]_lat[latitude]. npz”. Here, [var] is the abbreviation used in Table 3. The data can be read using Python. with np.load(filename.npz) as npz:

data=np.ma.MaskedArray(**npz)

## 3 Experimental Design, Materials, and Methods

### 3.1 NICAM

We used 30-year global climate simulation data produced using NICAM running on the K computer [3]. This model does not use cumulus convection parameterization, and clouds are calculated explicitly using a cloud microphysics scheme, NICAM Single-moment Water 6 [6]. The horizontal resolution was approximately 14 km and the number of vertical levels was 38. As the boundary conditions, the monthly mean SST and sea ice taken by HadISST1 [7] were used. The simulations were conducted under conditions similar to those of the Atmospheric Model Intercomparison Project (AMIP) [8] on a project with a time constraint and have not undergone operational updates.

Characteristic phenomena such as TCs, Madden–Julian oscillations, equatorial waves, Asian monsoons, and global precipitation have been well simulated [3]. The simulation data of the experiment include precipitation, OLR at the top-of-atmosphere, zonal and meridional wind, pressure, temperature, water vapor, and cloud substances (graupel, ice, liquid, rain, and snow) for 30 years since 1979. For further details, please see the review paper [9].

### 3.2 TC Tracking

To detect pre-TCs, TCs, and post-TCs, a TC tracking algorithm was employed for the 6-h outputs of zonal and meridional wind, SLP, and temperature. This algorithm was originally proposed by Sugi et al. [10] and tuned for the NICAM simulation data [2,3].

In the first step, candidates of the TC center, which are the grid points at which the SLP is 0.5 hPa less than the mean of its surrounding area, were searched. In the next step, these grid points were connected to their nearest neighbors in time to form a track such that criteria (i)–(vi) were satisfied:

(i) Maximum wind speed at the 10 m level of > 17.5 ms^−1^;
(ii) Maximum relative vorticity at 850 hPa of > 1.0  ×  10^−3^ s^−1^;
(iii) Sum of temperature deviations at 300, 500, and 700 hPa of > 2 K;
(iv) Wind speed at 850 hPa greater than that at 300 hPa;
(v) Duration of the TCs is > 36 h;
(vi) TC generated within 30° S–30° N.

Each track included the following life stages: pre-TC, TC, and post-TC. The genesis of a TC is referred to as the first step at which conditions (i)–(iv) are satisfied in at least six successive time steps. The track before genesis was regarded as that of the pre-TC. Once the four conditions were no longer satisfied after genesis, the track was categorized as post-TC.

## Ethics statements

*This work does not involve any studies with human or animal subjects.*

## CRediT authorship contribution statement

**Daisuke Matsuoka:** Conceptualization, Visualization, Writing – original draft, Writing – review & editing. **Chihiro Kodama:** Methodology, Software, Writing – review & editing. **Yohei Yamada:** Software, Validation, Writing – review & editing. **Masuo Nakano:** Methodology, Validation, Writing – review & editing.

## Declaration of Competing Interest

The authors declare that they have no known competing financial interests or personal relationships that could have appeared to influence the work reported in this paper.

## Data Availability

Dataset of Tropical Cyclone in a High-Resolution Global Nonhydrostatic Atmospheric Simulation (Original data) (Mendeley Data).
